# Supplementary material for: 3D multiplexed tissue imaging reconstruction and optimized region of interest (ROI) selection through deep learning model of channels embedding
Source: Front Bioinform. 2023 Oct 19;3:1275402. doi: 10.3389/fbinf.2023.1275402 (PMC10620917; doi:10.3389/fbinf.2023.1275402)
Supplement: Supplementary file 2 [file DataSheet1.pdf]

SUPPLEMENTARY INFORMATION

SUPPLEMENTARY FIGURES

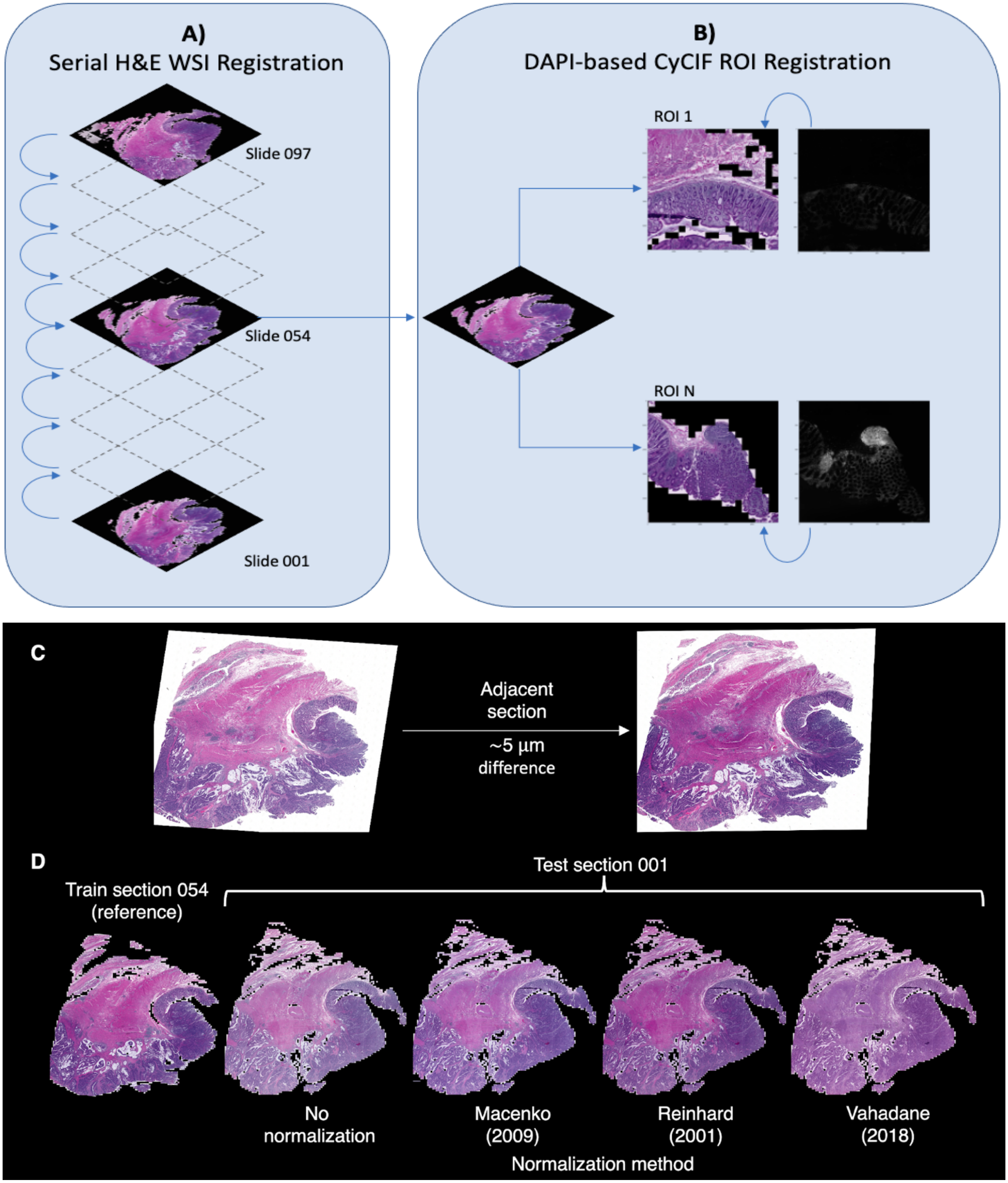

Supp. Figure 1: Registration schema for 3D H&E and CyCIF imaging dataset and normalization overview. (A) To register H&E in the three-dimensional setting, we sequentially registered all slides to the center using the transforms propagated from previous layers. (B) CyCIF was then finely registered to the adjacent H&E images at the ROI level to maximize single-cell level correspondence. Registration of CyCIF and H&E was performed using binarized DAPI and thresholded H&E to align nuclei. (C) Tissue sections are subject to technical variability in stain intensity, even between adjacent sections that are separated by only ~5 μm. (D) Representative results of H&E stain normalization. The stain intensity distribution of the test section 001 is transformed to match that of the reference section 054 which was used for SHIFT model training.

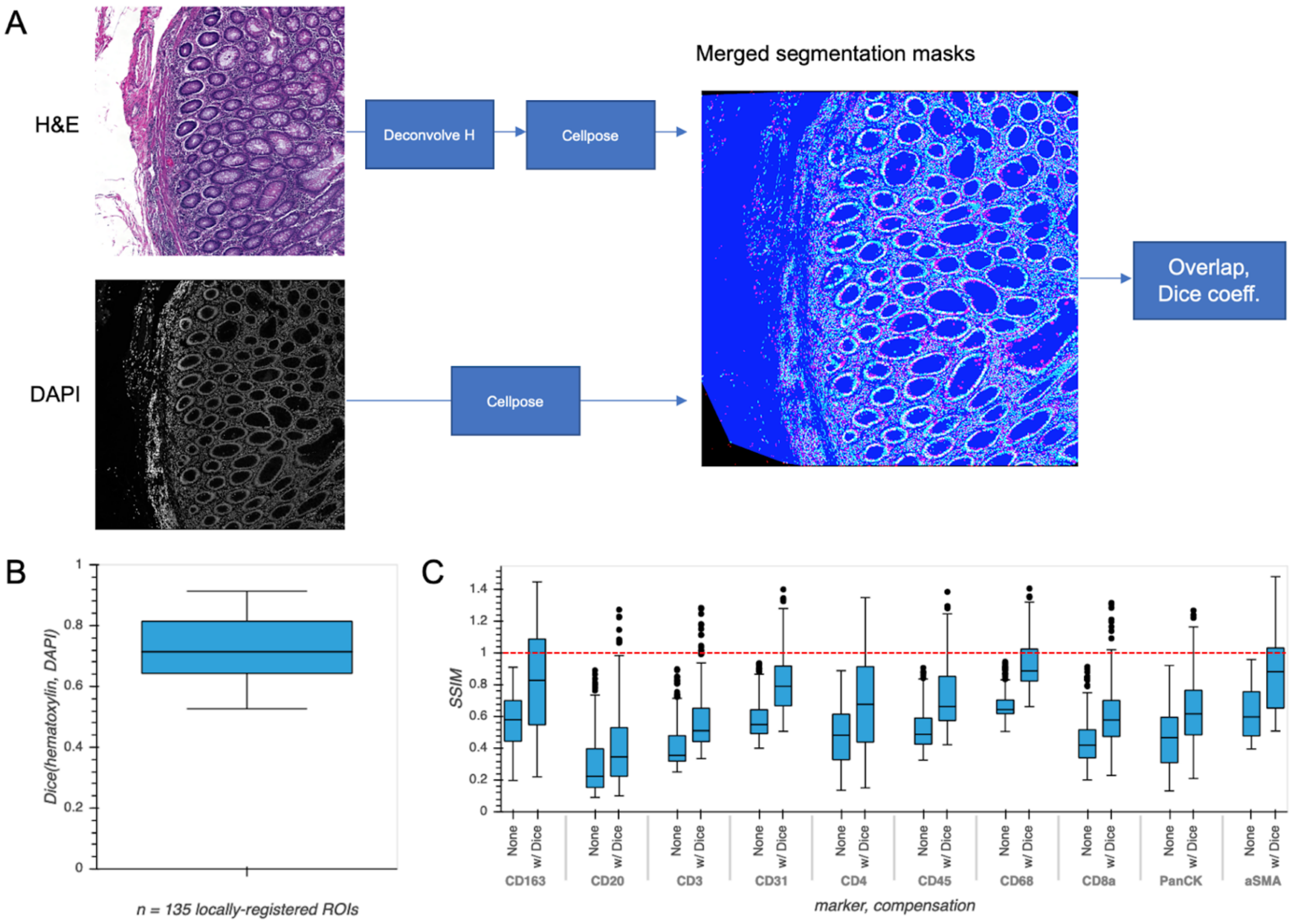

Supp. Figure 2: Estimating upper bound on SHIFT performance by measuring concordance between nuclei overlap in adjacent sections for locally-registered ROIs from H&E/CyCIF test sections 096/097. (A) The Dice coefficients describing the overlap of nuclear masks from ROIs of adjacent sections were used as compensation factors for evaluating virtual stains. (B) Boxplot describing the distribution of Dice coefficients of the 135 locally-registered ROIs from H&E/CyCIF test sections 096/097. (C) Boxplots describing the distributions of structural similarity (SSIM) of real vs. virtual CyCIF ROIs over the 135 locally-registered ROIs from H&E/CyCIF test sections. The red dotted line indicates the unity line describing Dice-compensated SSIM.

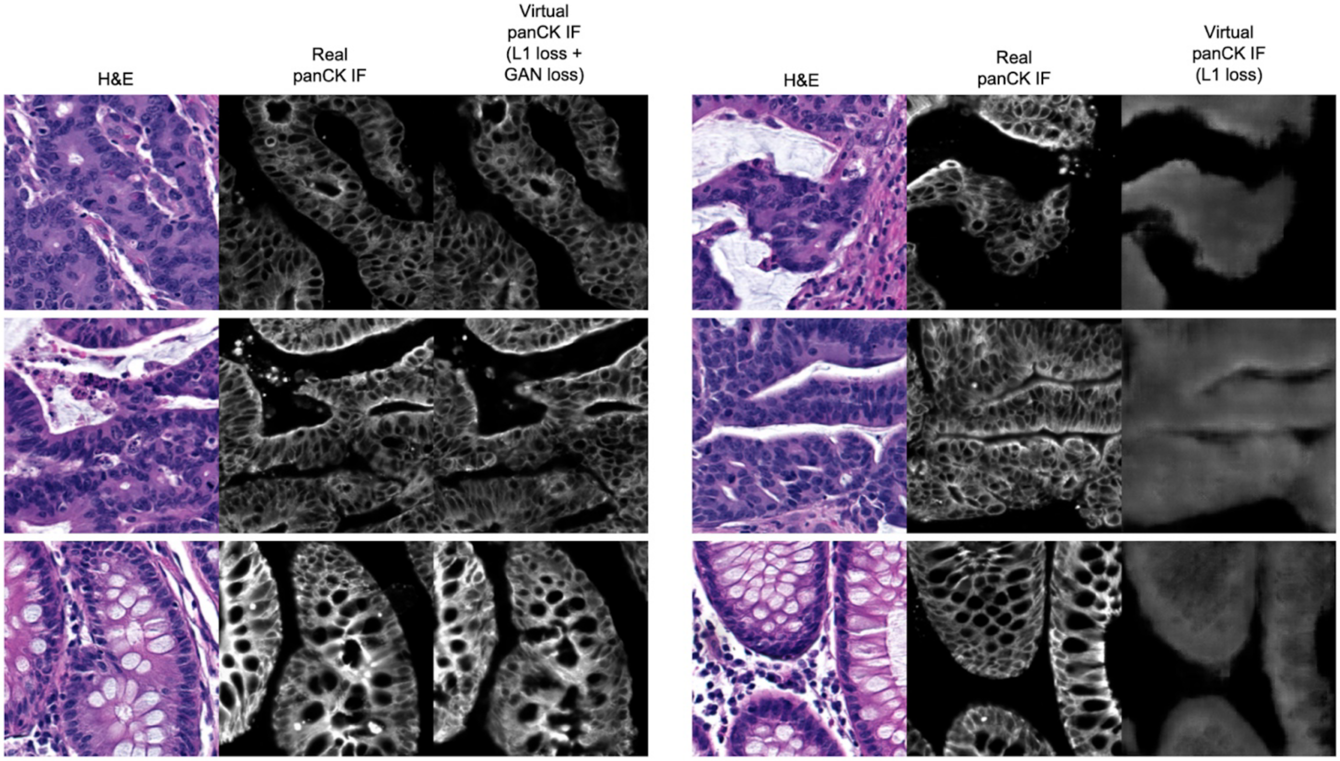

Supp. Figure 3: Virtual staining outcomes with different loss functions. The left panels correspond to results from the full SHIFT model (generator and discriminator) and the right panels correspond to results from a model consisting of a generator only.

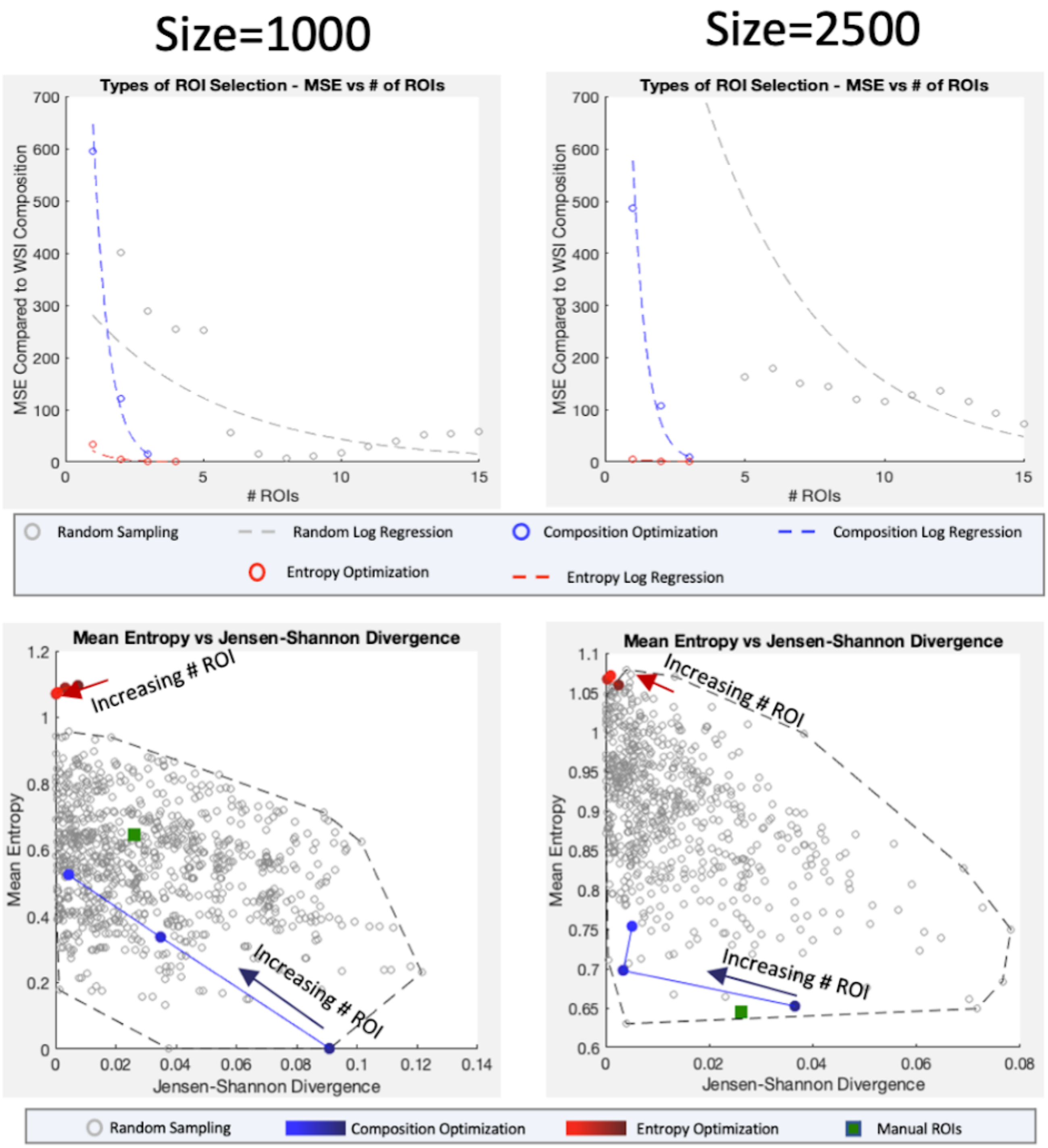
Size=1000
Size=2500
Types of ROI Selection - MSE vs # of ROIs
MSE Compared to WSI Composition
ROIs
Random Sampling
Random Log Regression
Composition Optimization
Composition Log Regression
Entropy Optimization
Entropy Log Regression
Mean Entropy vs Jensen-Shannon Divergence
Mean Entropy
Jensen-Shannon Divergence
Increasing # ROI
Random Sampling
Composition Optimization
Entropy Optimization
Manual ROIs

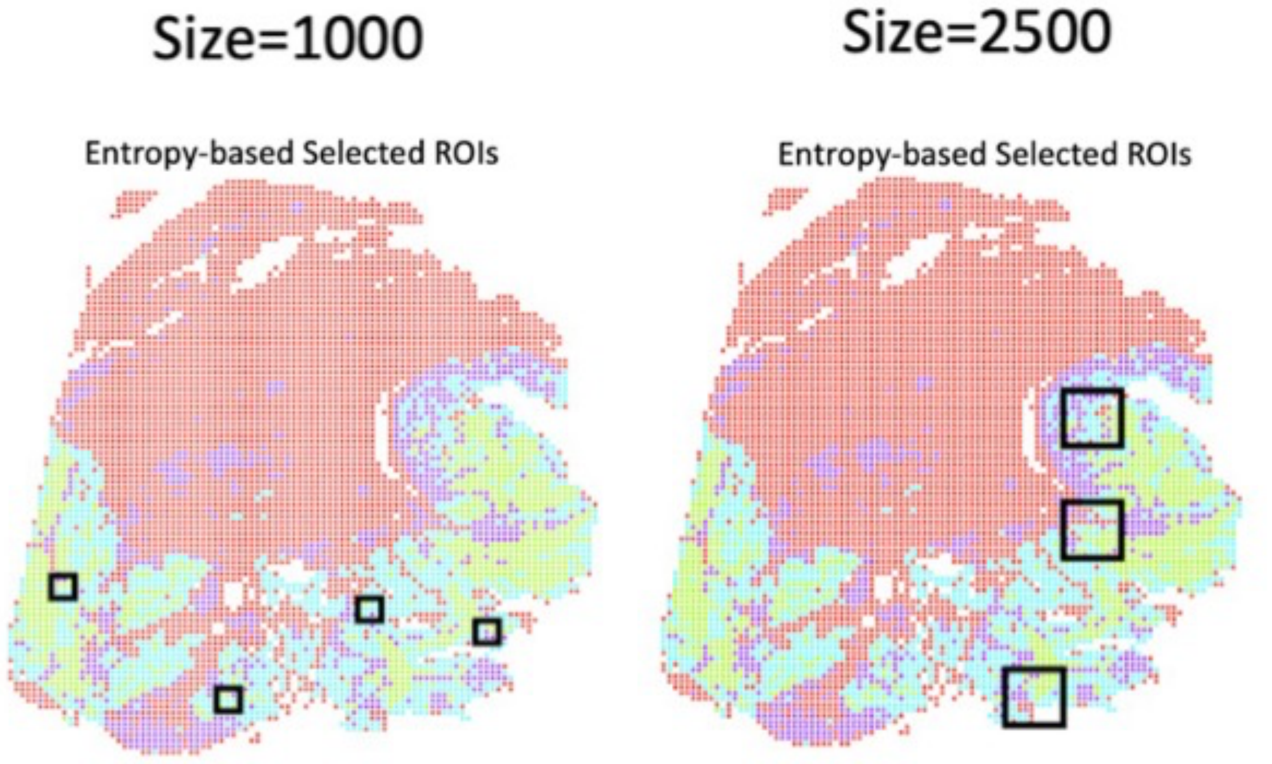

Supp. Figure 4: Optimization of ROI selection within a restricted set of cells. For two ROI sizes (1000 pixel, 2500 pixel) and three sampling techniques (random sampling, convex optimization using cell type composition, convex optimization using cell type composition, and regional entropy), we calculate the optimal selection of ROI. Before calculation, we restrict the cell types of interest to only tumor and immune type cells targeted by manual annotation. **(Top row)** By calculating the MSE for a range of ROI, we can evaluate each technique's rate and quality of convergence. (**Middle row)** Selections of representative ROIs are evaluated based on two metrics (Entropy for tissue heterogeneity and Jensen-Shannon Divergence for composition similarity.) Random sets of 7 ROIs are generated 1000 times to portray the baseline pattern. Selections from convex optimizations are plotted with increasing numbers of ROIs to show the change in performance. The performance of the manually selected ROIs is also shown to emphasize the bias in targeted sampling. **(Bottom row)** The optimal ROIs are shown for convex entropy optimization at each size of ROI. Image colors portray the XAE-labeled cell types (red being cell types not considered in this analysis).

SUPPLEMENTARY TABLES

Layer	Generator
D1	Conv2d(3, 64, kernel_size=(4,4), stride=(2,2), padding=(1,1), bias=False)<br>LeakyReLU(negative_slope=0.2, inplace=True)
D2	Conv2d(64, 128, kernel_size=(4,4), stride=(2,2), padding=(1,1), bias=False)<br>BatchNorm2d(128, eps=1e-05, momentum=0.1, affine=True)<br>LeakyReLU(negative_slope=0.2, inplace=True)
D3	Conv2d(128, 256, kernel_size=(4,4), stride=(2,2), padding=(1,1), bias=False)<br>BatchNorm2d(256, eps=1e-05, momentum=0.1, affine=True)<br>LeakyReLU(negative_slope=0.2, inplace=True)
D4	Conv2d(256, 512, kernel_size=(4,4), stride=(2,2), padding=(1,1), bias=False)<br>BatchNorm2d(512, eps=1e-05, momentum=0.1, affine=True)<br>LeakyReLU(negative_slope=0.2, inplace=True)
D5	Conv2d(512, 512, kernel_size=(4,4), stride=(2,2), padding=(1,1), bias=False)<br>BatchNorm2d(512, eps=1e-05, momentum=0.1, affine=True)<br>LeakyReLU(negative_slope=0.2, inplace=True)
D6	Conv2d(512, 512, kernel_size=(4,4), stride=(2,2), padding=(1,1), bias=False)<br>BatchNorm2d(512, eps=1e-05, momentum=0.1, affine=True)<br>LeakyReLU(negative_slope=0.2, inplace=True)
D7	Conv2d(512, 512, kernel_size=(4,4), stride=(2,2), padding=(1,1), bias=False)<br>BatchNorm2d(512, eps=1e-05, momentum=0.1, affine=True)<br>LeakyReLU(negative_slope=0.2, inplace=True)

D8	Conv2d(512, 512, kernel_size=(4,4), stride=(2,2), padding=(1,1), bias=False)<br>ReLU(inplace=True)
U1	ConvTranspose2d(512, 512, kernel_size=(4,4), stride=(2,2), padding=(1,1), bias=False)<br>BatchNorm2d(512, eps=1e-05, momentum=0.1, affine=True)<br>ReLU(inplace=True)
U2	ConvTranspose2d(1024, 512, kernel_size=(4,4), stride=(2,2), padding=(1,1), bias=False)<br>BatchNorm2d(512, eps=1e-05, momentum=0.1, affine=True)<br>ReLU(inplace=True)
U3	ConvTranspose2d(1024, 512, kernel_size=(4,4), stride=(2,2), padding=(1,1), bias=False)<br>BatchNorm2d(512, eps=1e-05, momentum=0.1, affine=True)<br>ReLU(inplace=True)
U4	ConvTranspose2d(1024, 512, kernel_size=(4,4), stride=(2,2), padding=(1,1), bias=False)<br>BatchNorm2d(512, eps=1e-05, momentum=0.1, affine=True)<br>ReLU(inplace=True)
U5	ConvTranspose2d(1024, 256, kernel_size=(4,4), stride=(2,2), padding=(1,1), bias=False)<br>BatchNorm2d(256, eps=1e-05, momentum=0.1, affine=True)<br>ReLU(inplace=True)
U6	ConvTranspose2d(512, 128, kernel_size=(4,4), stride=(2,2), padding=(1,1), bias=False)<br>BatchNorm2d(128, eps=1e-05, momentum=0.1, affine=True)<br>ReLU(inplace=True)
U7	ConvTranspose2d(256, 64, kernel_size=(4,4), stride=(2,2), padding=(1,1), bias=False)

	BatchNorm2d(64, eps=1e-05, momentum=0.1, affine=True)<br>ReLU(inplace=True)
U8	ConvTranspose2d(128, 1, kernel_size=(4,4), stride=(2,2), padding=(1,1))<br>Tanh()
Layer	Discriminator
1	Conv2d(4, 64, kernel_size=(4,4), stride=(2,2), padding=(1,1))<br>LeakyReLU(negative_slope=0.2, inplace=True)
2	Conv2d(64, 128, kernel_size=(4,4), stride=(2,2), padding=(1,1), bias=False)<br>BatchNorm2d(128, eps=1e-05, momentum=0.1, affine=True)<br>LeakyReLU(negative_slope=0.2, inplace=True)
3	Conv2d(128, 256, kernel_size=(4,4), stride=(2,2), padding=(1,1), bias=False)<br>BatchNorm2d(256, eps=1e-05, momentum=0.1, affine=True)<br>LeakyReLU(negative_slope=0.2, inplace=True)
4	Conv2d(256, 512, kernel_size=(4,4), stride=(1,1), padding=(1,1), bias=False)<br>BatchNorm2d(512, eps=1e-05, momentum=0.1, affine=True)<br>LeakyReLU(negative_slope=0.2, inplace=True)
5	Conv2d(512, 1, kernel_size=(4,4), stride=(1,1), padding=(1,1))

Supplementary Table 1: architecture of SHIFT models.

Layer	Encoders	Shared?
1	ReflectionPad2d((3, 3, 3, 3))<br>Conv2d(3, 64, kernel_size=(7,7), stride=(1,1))<br>InstanceNorm2d(64, eps=1e-05, momentum=0.1, affine=False)<br>LeakyReLU(negative_slope=0.2, inplace=True)	No
2	Conv2d(64, 128, kernel_size=(4,4), stride=(2,2), padding=(1,1))<br>InstanceNorm2d(128, eps=1e-05, momentum=0.1, affine=False)<br>ReLU(inplace=True)	No
3	Conv2d(128, 256, kernel_size=(4,4), stride=(2,2), padding=(1,1))<br>InstanceNorm2d(256, eps=1e-05, momentum=0.1, affine=False)<br>ReLU(inplace=True)	No
4	ResBlock(N=256 ,K=3, S=1)	No
5	ResBlock(N=256, K=3, S=1)	No
6	ResBlock(N=256, K=3, S=1)	No
z	ResBlock(N=256, K=3, S=1)<br>Reparameterization()	Yes
Layer	Decoders	Shared?
1	ResBlock(N=256, K=3, S=1)	Yes
2	ResBlock(N=256, K=3, S=1)	No
3	ResBlock(N=256, K=3, S=1)	No
4	ResBlock(N=256, K=3, S=1)	No
5	ConvTranspose2d(256, 128, kernel_size=(4,4), stride=(2,2), padding=(1,1))<br>InstanceNorm2d(128, eps=1e-05, momentum=0.1, affine=False)	No

	LeakyReLU(negative_slope=0.2, inplace=True)	
6	ConvTranspose2d(128, 64, kernel_size=(4,4), stride=(2,2), padding=(1,1))<br>InstanceNorm2d(64, eps=1e-05, momentum=0.1, affine=False)<br>LeakyReLU(negative_slope=0.2, inplace=True)<br>ReflectionPad2d((3, 3, 3, 3))	No
7	Conv2d(64, 3, kernel_size=(7,7), stride=(1,1))<br>Tanh()	No
Layer	Discriminators	Shared?
1	Conv2d(11, 64, kernel_size=(4, 4), stride=(2, 2), padding=(1, 1))<br>LeakyReLU(negative_slope=0.2, inplace=True)	No
2	Conv2d(64, 128, kernel_size=(4, 4), stride=(2, 2), padding=(1, 1))<br>InstanceNorm2d(128, eps=1e-05, momentum=0.1, affine=False)<br>LeakyReLU(negative_slope=0.2, inplace=True)	No
3	Conv2d(128, 256, kernel_size=(4, 4), stride=(2, 2), padding=(1, 1))<br>InstanceNorm2d(256, eps=1e-05, momentum=0.1, affine=False)<br>LeakyReLU(negative_slope=0.2, inplace=True)	No
4	Conv2d(256, 512, kernel_size=(4, 4), stride=(2, 2), padding=(1, 1))<br>InstanceNorm2d(512, eps=1e-05, momentum=0.1, affine=False)<br>LeakyReLU(negative_slope=0.2, inplace=True)	No
5	Conv2d(512, 1, kernel_size=(3, 3), stride=(1, 1), padding=(1, 1))	No
	ResBlock	
	ReflectionPad2d((1, 1, 1, 1))<br>Conv2d(N, N, kernel_size=(K, K), stride=(S, S))<br>InstanceNorm2d(N, eps=1e-05, momentum=0.1, affine=False)<br>ReLU(inplace=True)<br>ReflectionPad2d((1, 1, 1, 1))	

Conv2d(N, N, kernel_size=(K, K), stride=(S, S))<br>InstanceNorm2d(N, eps=1e-05, momentum=0.1, affine=False)

Supplementary Table 2: architectures of XAE models.
